# Emotional cues reduce Pavlovian interference in feedback-based go and nogo learning

**DOI:** 10.1007/s00426-024-01946-9

**Published:** 2024-03-14

**Authors:** Julian Vahedi, Annakarina Mundorf, Christian Bellebaum, Jutta Peterburs

**Affiliations:** 1 Faculty of Mathematics and Natural Sciences, Heinrich Heine University Düsseldorf, Düsseldorf, Germany; 2 Institute for Systems Medicine, Department of Human Medicine, MSH Medical School, Hamburg, Germany

## Abstract

It is easier to execute a response in the promise of a reward and withhold a response in the promise of a punishment than vice versa, due to a conflict between cue-related Pavlovian and outcome-related instrumental action tendencies in the reverse conditions. This robust learning asymmetry in go and nogo learning is referred to as the Pavlovian bias. Interestingly, it is similar to motivational tendencies reported for affective facial expressions, i.e., facilitation of approach to a smile and withdrawal from a frown. The present study investigated whether and how learning from emotional faces instead of abstract stimuli modulates the Pavlovian bias in reinforcement learning. To this end, 137 healthy adult participants performed an orthogonalized Go/Nogo task that fully decoupled action (go/nogo) and outcome valence (win points/avoid losing points). Three groups of participants were tested with either emotional facial cues whose affective valence was either congruent (CON) or incongruent (INC) to the required instrumental response, or with neutral facial cues (NEU). Relative to NEU, the Pavlovian bias was reduced in both CON and INC, though still present under all learning conditions. Importantly, only for CON, the reduction of the Pavlovian bias effect was adaptive by improving learning performance in one of the conflict conditions. In contrast, the reduction of the Pavlovian bias in INC was completely driven by decreased learning performance in non-conflict conditions. These results suggest a potential role of arousal/salience in Pavlovian-instrumental regulation and cue-action congruency in the adaptability of goal-directed behavior. Implications for clinical application are discussed.

**Supplementary Information:**

The online version contains supplementary material available at 10.1007/s00426-024-01946-9.

## Introduction

In order to optimize our behavior during decision-making, we have to select actions that most likely lead to favorable outcomes and avoid actions resulting in unfavorable consequences. Thus, adaptive behavior requires integrating our past experiences with actions and their consequences to maximize rewards and minimize losses in the future. Decision-making in everyday life often requires choosing between executing a specific action (*go*) and withholding an action (*nogo*). For instance, when standing at a busy intersection, we would ideally cross the road only when no vehicles are approaching, thus stop and wait until the road is empty and can be safely crossed.

Early behaviorist theories predicted that individuals generally align their behavior to the best outcome (e.g. Thorndike, 1927). Notably, not all contingencies between (in)actions and their consequences are learned equally well. Under the influence of Pavlovian learning, associations between cues and their expected outcomes seem to trigger actions in a stereotyped and valence-dependent manner: the expectation of reward facilitates action invigoration, whereas the expectation of punishment facilitates the inhibition of action, which has been referred to as *Pavlovian bias* in the literature (see e.g. Cavanagh et al., 2013; Guitart-Masip et al., 2011, 2012, 2014a; Peterburs et al., 2021, 2022).

The Pavlovian bias has been typically studied using a feedback-based Go/Nogo learning task first described by Guitart-Masip et al. (2011). This task orthogonalizes action and outcome valence, resulting in four experimental conditions: pressing a button in order to obtain a reward (go to win), pressing a button in order to avoid punishment (go to avoid-losing), withholding a button press in order to obtain a reward (nogo to win), and withholding a button press in order to avoid punishment (nogo to avoid-losing). Go to avoid-losing and nogo to win are often referred to as conflict conditions, while go to win and nogo to avoid-losing are referred to as non-conflict conditions (e.g. Albrecht et al., 2016; Cavanagh et al., 2013). Participants’ learning performance is typically better for non-conflict relative to conflict conditions, i.e., better for go to win compared to go to avoid-losing, and for nogo to avoid-losing compared to nogo to win. This asymmetric coupling of action and outcome valence reflecting Pavlovian learning biases seems to be quite robust and has been repeatedly demonstrated in a variety of experimental studies (e.g. Cavanagh et al., 2013; Guitart-Masip et al., 2011; Guitart-Masip et al., 2012; Peterburs et al., 2021).

Crucially, action selection is not fully determined by either instrumental or Pavlovian learning but rather from a combination of both (Dorfman & Gershman, 2019; O’Doherty, 2016). Instrumental control of action based on learning from performance feedback is rather slow but highly flexible as it enables goal-directed behavior. In contrast, Pavlovian control of action is fast and reflexive but also more rigid. Hence, the Pavlovian bias might be particularly useful when the Pavlovian responses are in alignment with the required instrumental response (e.g., approaching food), but can hinder optimal decision-making when Pavlovian responses conflict with the required instrumental response. In evolutionary terms, automatic, prepotent Pavlovian responses might have been advantageous, particularly in new and uncertain environments, because they served as a quickly available decision heuristic (Boureau et al., 2015; Dayan & Seymour, 2009; Rangel et al., 2008). Nonetheless, to ensure adaptive behavior, both instrumental and Pavlovian learning systems need to be carefully balanced.

Accordingly, Pavlovian bias abnormalities have been linked to maladaptive behaviors including impulsivity (Eisinger et al., 2020) and are associated with numerous (neuro-)psychiatric conditions, including anxiety (Peterburs et al., 2022) and traumatic stress (Ousdal et al., 2018), schizophrenia (Albrecht et al., 2016), and Parkinson’s disease (Eisinger et al., 2020; Wagenbreth et al., 2015). For depression, results have been inconsistent, with Nord et al. (2018) reporting enhanced and Moutoussis et al. (2018b) reporting intact Pavlovian biases. Differences have also been reported for different age groups, indicating that adolescents exhibit reduced Pavlovian control over action compared with children and young adults, possibly facilitating exploration and openness to new experiences (Raab & Hartley, 2020). Interestingly, as age progresses in adulthood, Pavlovian biases have been found to decrease again, with the effect particularly driven by a decrease in Pavlovian facilitation of action in the promise of reward (Chen et al., 2018). Note, however, that Betts et al. (2019) found no modulatory effect of age on the Pavlovian bias but increased bias towards action in children and adolescents and decreased reward and punishment sensitivity in midlife and older adults. Beyond that, higher IQ has been shown to be associated with weaker Pavlovian influence (Moutoussis et al., 2018a).

The neural source of the Pavlovian bias has often been linked to the striatum along with its key neuromodulator dopamine (de Boer et al., 2019; Guitart-Masip et al., 2011, 2012; Richter et al., 2014; Swart et al., 2017). However, results of Guitart-Masip et al. (2014b) indicated a reduced Pavlovian bias after boosting dopamine levels using levodopa that could not be attributed to striatal dopaminergic activity, but was rather explained by a potential involvement of the prefrontal cortex in overcoming Pavlovian biases. This assumption was supported by findings of Cavanagh et al. (2013) who could show that midfrontal oscillatory theta power was associated with greater ability to overcome the Pavlovian biases on the inter- as well as intraindividual level, indicating that prefrontal control mechanisms are capable of resolving conflicting action requirements. In line with that, a recent study by Kim et al. (2023) provided causal evidence for a prefrontal role in overcoming Pavlovian-instrumental conflicts by showing reduced Pavlovian bias after anodal transcranial direct current stimulation over the dorsolateral prefrontal cortex.

Recent investigations have focused on how Pavlovian influences on instrumental learning can be manipulated or overcome. Motivated by findings indicating a reduced role of the striatum in observational learning (e.g. Bellebaum et al., 2012; Kobza et al., 2012), Peterburs et al. (2021) compared learning performance in a modified orthogonalized Go/Nogo task among active and observational learners, but found comparable Pavlovian bias in both groups. Similarly, the Pavlovian bias was hardly modifiable in a recent series of experiments by Ereira et al. (2021). Here, five groups of participants underwent three days of training in different variants of an orthogonal Go/Nogo task that varied in the response domain (motoric vs. semantic), in stimulus presentation (single vs. massed), and in gamification (no gamification vs. gamification). All subjects underwent a consecutive test session on the third day of training. Only in the semantic task version with massed stimulus presentation and gamification a training-induced reduction of the Pavlovian bias, that even persisted in an independent task, could be observed. The Pavlovian bias remained robust under all other training conditions.

In the standard orthogonalized Go/Nogo task, neutral cues, often abstract visual stimuli such as fractal images, are used which are then assigned positive or negative valence through the process of learning (Guitart-Masip et al., 2011, 2012; Peterburs et al., 2021). However, for emotional-affective stimuli, valence does not require previous learning. Valence-action biases similar to the Pavlovian bias in reinforcement learning have been assumed for the emotional domain, with positive emotional valence favoring response invigoration and negative emotional valence favoring withdrawal behavior (Cacioppo et al., 1999; Lang, 1995). This contingency between valenced emotional stimuli and approach/avoidance has been supported by several studies (e.g. Chen & Bargh, 1999; Krieglmeyer et al., 2010; Phaf et al., 2014; Seibt et al., 2008; Seidel et al., 2010). Faster response times (RTs) have been reported for congruent valence-action pairings, i.e., when approaching positive emotional stimuli and avoiding negative emotional stimuli, compared with incongruent valence-action pairings, i.e., when approaching negative emotional stimuli and avoiding positive emotional stimuli (Krieglmeyer et al., 2010; Phaf et al., 2014; Seidel et al., 2010). However, it must be noted that these findings were based on studies in which participants were required to show an active response for both approach and avoidance. Emotion effects on action execution and action suppression have been typically investigated using emotional variants of Go/Nogo tasks (e.g. Schrammen et al., 2020), reporting more commission errors to positive than to negative facial stimuli as well as increased RTs to negative compared to positive facial stimuli (Hare et al., 2005; Schulz et al., 2007).

Whether and how emotional valence affects the Pavlovian bias has not been fully answered yet. Asci et al. (2019) studied the effects of task-irrelevant emotional background stimuli on action-valence compatibility effects in an equiprobable Go/Nogo task. However, while there was evidence for reward-related facilitation of action over inaction, the authors did not find any modulatory effect of emotional background. In a more recent study, Weber et al. (2022) studied the effect of video-based induction of positive and negative affect on Pavlovian biases. Strikingly, the results revealed that induced affect did not have an effect on overall approach or avoidance tendencies, and therefore did not modulate the Pavlovian bias.

However, both of these studies used emotional manipulations with rather low salience, as emotional stimuli or affect were introduced in a task-irrelevant manner. Rotteveel and Phaf (2004) suggested that affective cues have to be consciously processed in order to bias action tendencies. Beyond that, it should be noted that emotional stimuli and induced affect do not necessarily affect approach and avoidance behavior in the same direction. While negative emotional stimuli such as angry faces are generally associated with avoidance behavior, induced negative affect, especially experienced anger, might be more strongly related to approach behavior (Carver & Harmon-Jones, 2009; Harmon-Jones, 2003).

Taking this into account, using emotional stimuli and implementing emotional valence more saliently, e.g., by using emotional instead of neutral cues, may be better suited to uncover emotionality effects on the Pavlovian bias. In this context, emotional faces may be of particular interest as facial expressions play a vivid role in social interactions. A smile, i.e., a happy face, automatically evokes approach, while a frown, i.e., an angry face, triggers withdrawal behavior (Marsh et al., 2005; Nikitin & Freund, 2019; Seidel et al., 2010). Facial stimuli have been successfully used as feedback stimuli in a social task variant of the orthogonalized Go/Nogo task, indicating that emotional faces are capable of signaling reward and punishment (Thompson & Westwater, 2017). There is also evidence that brain areas activated by the perception of happy faces overlap with reward-related areas of the basal ganglia (Chakrabarti et al., 2006), which, depending on whether the emotional valence matches or opposes the required instrumental response, may facilitate or hinder learning.

The aim of the present study was to investigate whether the Pavlovian bias can be modulated by social affective cues in an emotional variant of the orthogonalized Go/Nogo task. In this task, facial stimuli were used as Pavlovian cues (as opposed to social feedback). Previous findings indicate that non-conflict conditions (i.e., go to win and nogo to avoid-losing) are already relatively easy to learn, with at least go to win often resulting in a ceiling effect (e.g., see Peterburs et al., 2022). Therefore, we hypothesized emotional manipulations to have an asymmetric effect on task performance, expecting a greater influence on conflict conditions (i.e., go to avoid-losing and nogo to win) relative to non-conflict conditions. In particular, go to win may only be hardly modifiable (if at all). In contrast, whether and how emotional manipulations will affect task performance for nogo to avoid-losing is more speculative. On the one hand, task performance may be hardwired and resistant to emotional manipulations, as assumed for go to win. On the other hand, nogo to avoid-losing could be susceptible to emotional manipulations in the same way as is supposed for conflict conditions.

Consequently, we hypothesized that learning from a facial cue whose motivational prospect was congruent with the required instrumental action (i.e., emotional-congruent; happy face for go response, angry face for nogo response) should facilitate learning by particularly boosting performance in conflict conditions (go to avoid-losing and nogo to win). As a result, the Pavlovian bias should decrease or even disappear compared to a task version with neutral facial cues. In contrast, we expected that learning from facial cues whose motivational prospect was incongruent with the required instrumental action (i.e., emotional-incongruent; happy face for nogo response, angry face for go response) should hinder learning. However, whether and how this manipulation affects the Pavlovian bias is more speculative. As assumed for the emotional-congruent learning condition, learning may be relatively unaffected in the non-conflict conditions (go to win, nogo to avoid-losing), due to the relative ease and robustness of learning. For go to avoid-losing and nogo to win, learning performance should decrease. Thus, the Pavlovian bias should be enhanced compared to a task version with neutral facial cues. Alternatively, it is also conceivable that learning performance will be equally impaired in all conditions, resulting in reduced learning performance overall but comparable Pavlovian bias relative to the neutral task version. On a statistical level, the hypothesized modulation of the Pavlovian bias would correspond to an interaction effect between the factors learning condition, required action and outcome valence.

Last, in an exploratory analysis, we investigated a potential link between the Pavlovian bias and reward and punishment sensitivity, as individual differences in reward and punishment have shown to be associated with striatal activation during reward and avoidance learning (Kim et al., 2015). Reward and punishment sensitivity were measured via self-report using the behavioral inhibition system/behavioral activation system (BIS/BAS) scales (Carver & White, 1994).

## Materials and methods

### Participants

Sample size was roughly estimated based on previous studies that employed the orthogonalized Go/Nogo task which collected data from between around 20 and 50 participants (e.g. Cavanagh et al., 2013; Ereira et al., 2021; Guitart-Masip et al., 2012; Peterburs et al., 2021). Thus, we aimed at a sample size of approximately 40 participants per experimental group (after exclusions), which we assumed to have sufficient statistical power to detect potential effects related to our manipulations. Consequently, a total of 137 young and healthy volunteers participated in the study (see Table S1) that were recruited at two test sites, Heinrich-Heine University Düsseldorf, Germany (HHU; *n* = 66), and MSH Medical School Hamburg, Germany (MSH; *n =* 71) by public advertisement and/or social media. At each test site, participants were randomly assigned to one of three experimental groups: emotional-congruent (CON; *n* = 43), emotional-incongruent (INC; *n* = 48), or neutral (NEU; *n* = 46).

Importantly, a simulation-based sensitivity power analysis conducted in R using the *simr* package (Green & MacLeod, 2016) based on the final sample size (i.e. after exclusions) attested sufficient statistical power (= 80%) to detect an (unstandardized) effect size of β = 25.3 for the three-way interaction between the factors learning condition, action and valence, used to operationalize the modulation of the Pavlovian bias effect by emotional cues (see Figure S1). Details on the sensitivity power analysis can be found in the Supplementary Material.

Participants were only eligible for participation if they reported no history of psychiatric or neurological disorders and were currently not taking any psychotropic medication. All participants had normal or corrected-to normal vision and were naïve to the study’s intent. Written informed consent was obtained from all participants prior to their participation. The study procedures conformed to the Declaration of Helsinki and had received ethical clearance by the Ethics Board of the Faculty of Mathematics and Natural Sciences at Heinrich-Heine-University, Germany, and the Ethics Board of the MSH Medical School Hamburg, Germany.

IQ estimates were obtained from each participant using a short multiple-choice vocabulary test (Mehrfachwahl-Wortschatz-Test B, MWT-B; Merz et al., 1975), consisting of 37 items. Each item contained a row of five words and participants had to correctly identify the real word among four pseudowords. Using norm tables, the sum scores were translated into IQ estimates, which have been shown to correlate fairly high with global IQ scores obtained by more elaborate intelligence tests (Lehrl et al., 1995). Participants also completed BIS/BAS scales (Carver & White, 1994; German version: Strobel et al., 2001). This 20-item self-report questionnaire assesses the dispositional sensitivity to punishment (BIS scale; 7 items) and reward (BAS scale; 13 items) based on Gray’s *Reinforcement Sensitivity Theory* (Gray, 1982, 1987). Note that the present study only used BIS and BAS total scores, as the German version does not confirm the full four dimensional structure of the original BIS/BAS scales (Strobel et al., 2001).

Data exclusion (for details, see Data Analysis) left a sample of 121 participants (CON: *n* = 40; INC: *n* = 40; NEU: *n* = 41) for statistical analyses (see Table 1). There were no differences between experimental groups with respect to age (*p* = .649), verbal IQ (*p* = .525) and BIS (*p* = .967). However, BAS scores did differ between experimental groups (*p* = .047), with slightly higher BAS scores in INC relative to CON (*p =* .041) but no difference between CON and NEU (*p* = .535) and INC and NEU (*p* = .736).

**Table 1:** Sample characteristics after exclusions

	CON (*n* = 40)	INC (*n* = 40)	NEU (*n* = 41)
Demographic characteristics			
mean (*SD*) age in years	22.08 (3.06)	21.95 (2.64)	22.56 (3.59)
sex (female/male), *n*	27/13	27/13	28/13
handedness (left/right/n.a.), *n*	6/34/0	3/34/3	2/36/3
Mean (*SD*) score			
verbal IQ	101.45 (11.69)	103.95 (9.96)	103.59 (10.14)
BIS	20.45 (4.11)	20.25 (3.94)	20.37 (3.75)
BAS	39.23 (5.88)	41.85 (3.82)	40.63 (4.10)

*Note.* Demographic data and questionnaire scores are provided. Abbreviations are used as follows: CON = emotional-congruent group, INC = emotional-incongruent group, NEU = neutral group, SD = standard deviation, n.a. = not available; BIS = Behavioral Inhibition System, BAS = Behavioral Activation System

### Experimental task

**Fig. 1:**
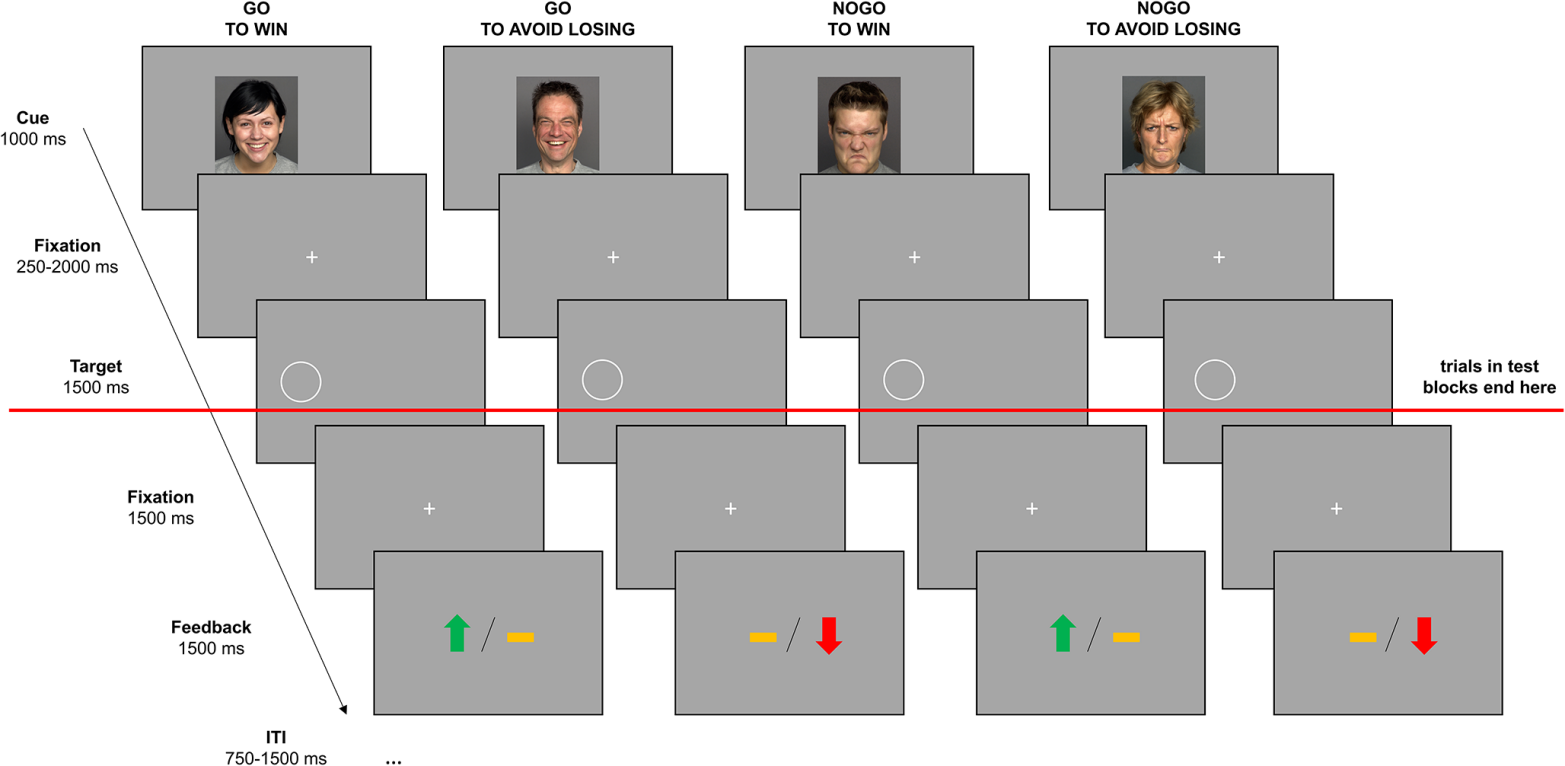
Experimental paradigm. Each trial started with one of four possible facial cues indicating one of the four experimental conditions (go to win, go to avoid losing, nogo to win, nogo to avoid losing). After a variable fixation phase, a white circle appeared either on the left or right side of the screen and participants were required to decide to either press the button corresponding to the side on which the circle appeared (go) or withhold the button press (nogo). After a short delay, symbolic feedback was provided: a green upward pointing arrow indicated winning ten points, a red downward pointing arrow indicated losing ten points, and a yellow horizontal bar indicated a draw (no points lost or won). Note that the task contained two block types, training and test blocks. Feedback was only provided in training blocks but not in test blocks. Facial cues were obtained from FACES database (Ebner et al., 2010). Note that this figure contains facial stimuli that are publicly available via the FACES platform. These faces do not correspond to the stimuli used in this study, except for their valence assignment

**Fig. 2:**
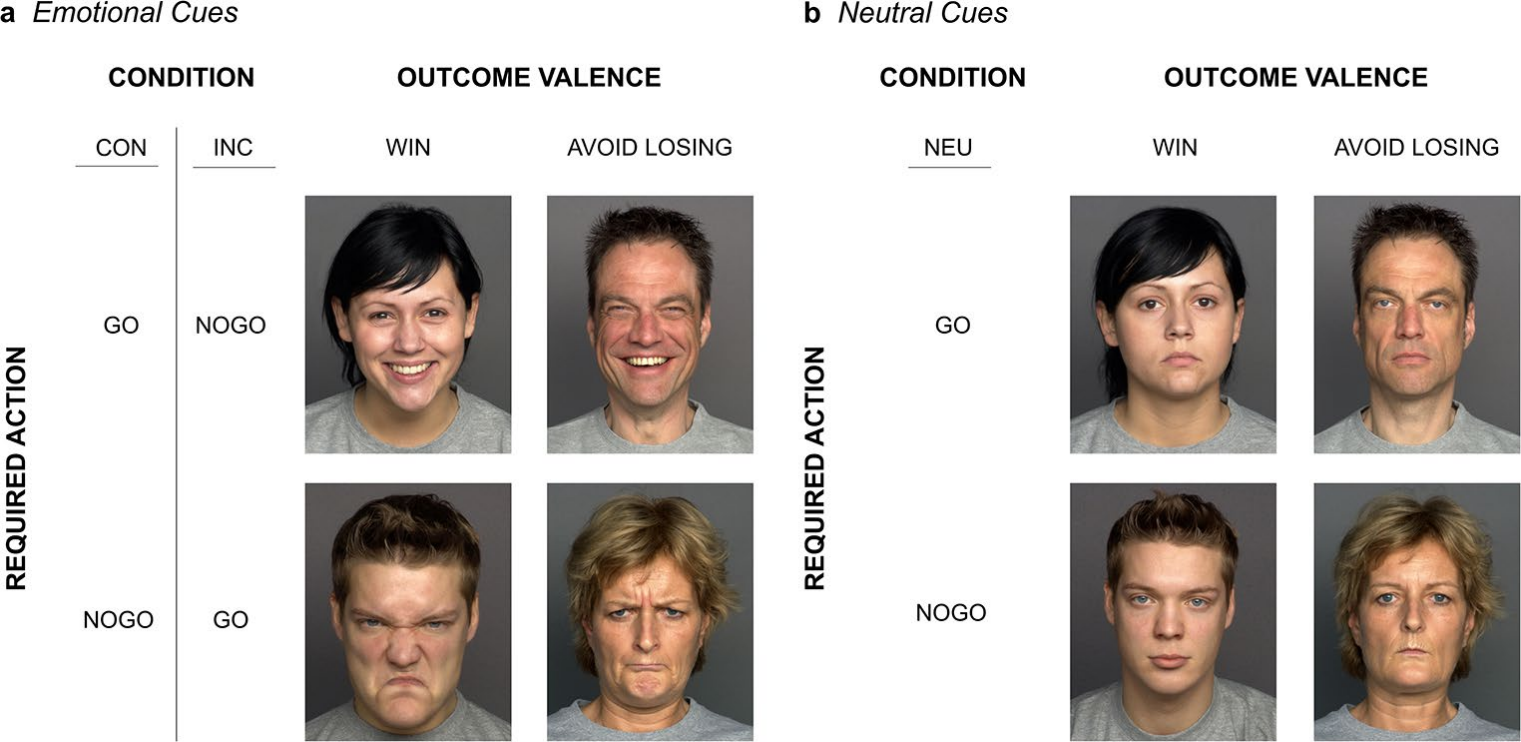
Facial stimuli were obtained from FACES data base (Ebner et al., 2010) and consisted of two sets (**a** and **b**) of photographs of four young adults (two females, two males). (**a**) shows the cues used for the two emotional learning conditions. For the emotional-congruent learning condition (CON), cues showing happy face expressions were used for the go conditions and cues showing angry face expressions were used for the nogo conditions. For the emotional-incongruent learning condition (INC), cues showing happy face expressions were used for the nogo conditions and cues showing angry face expressions were used for the go conditions. (**b**) shows the facial cues used for the neutral learning condition. Note that this figure contains facial stimuli that are publicly available via the FACES platform. These faces do not correspond to the stimuli used in this study, except for their valence assignment

Participants performed an emotional variant of the orthogonalized Go/Nogo task first described by Guitart-Masip et al. (2011). In this task, action (*go*, *nogo*) and outcome valence (*win*, *avoid-losing*) are fully decoupled in a balanced two-by-two design, resulting in four experimental conditions: go to win (GW), go to avoid-losing (GAL), nogo to win (NGW), and nogo to avoid-losing (NGAL). Figure 1 illustrates the time course and sequence of stimulus presentation in one trial of the task for these four experimental conditions. Each trial started with the presentation of a specific learning cue, i.e., one out of four facial stimuli for each experimental condition. For participants assigned to CON, the affective valence of the facial cue *matched* the associated action tendency that was required in that specific condition, i.e., GW and GAL were each indicated by a happy face, and NGW and NGAL were each indicated by an angry face. For participants assigned to INC, the affective valence of the facial cue *contradicted* the associated action tendency that was required in that specific condition, i.e., GW and GAL were each indicated by an angry face, and NGW and NGAL were each indicated by a happy face. Neutral faces served as learning cues for participants in the neutral learning condition. Stimulus assignment depending on learning condition is visualized in Fig. 2.

Each facial cue was displayed for 1000 milliseconds (ms), followed by a fixation cross for 250 to 2000 ms. Subsequently, an open white circle (target stimulus) was presented either on the left or right side of the screen for 1000 ms. Participants had to decide between responding (*go*) or not responding (*nogo*), and in case of choosing to respond, pressing the response button that indicated the location of the open circle (i.e., pressing the left/right CTRL button on a standard USB-keyboard when the open circle had appeared on the left/right). Button presses had to be made within 1000 ms. If participants chose not to respond, they had to simply let the response period pass. If participants accidentally pressed the wrong button, opposite to the actual side of the open circle, the trial was aborted and participants received explicit feedback that an invalid response had been made. When participants had made a valid decision, i.e., they had either indicated the correct side of the open circle or had not responded at all, another fixation cross was displayed for 1000 ms before symbolic feedback was provided. A green upward pointing arrow indicated that participants gained ten points (*win*), a red downward pointing arrow indicated that participants lost ten points (*loss*), and a yellow horizontal bar indicated that points had neither been gained nor lost (*draw*). Based on this performance feedback, participants could learn which action (*go*, *nogo*) for which learning cue was most likely associated with which outcome (*win*, *draw*, *loss*). Participants were explicitly instructed to use this performance feedback to maximize gains and minimize losses.

Importantly, for each learning cue, there was one favorable and one unfavorable outcome. In GW and NGW trials, the favorable outcome was to gain points (*win*) and the unfavorable outcome was to neither win nor lose points (*draw*). In GAL and NGAL trials, the favorable outcome was to avoid losing points (*draw*) and the unfavorable outcome was to lose points (*loss*). Responses that were associated with the best possible outcome in a given trial were classified as correct responses. Importantly, correct responses led to the favorable outcome in 80% of trials, while in the other 20%, participants received the unfavorable outcome (analogously, incorrect responses also led to favorable outcomes in 20% of trials, while in the other 80%, participants were presented the unfavorable outcome).

In total, the task comprised eight experimental blocks of 40 trials (ten for each trial type), amounting to 320 trials in total. Trial order was randomized within blocks. Following the procedures used in previous studies (Peterburs et al., 2021, 2022), there were two types of blocks, training blocks and test blocks, that were presented in alternate order. Training blocks and test blocks were nearly identical, except that in in training blocks, participants received performance-related symbolic feedback for each decision (as described above), while in test blocks, trials ended after the participant’s choice, thus no feedback was presented. Between blocks, participants could take a short rest and were informed about their current score. Unlike in the original task, described by Guitart-Masip et al. (2011), and following Peterburs et al. (2022), we implemented test blocks without feedback in order to assess the current state of learning that was not influenced by trial-by-trial adjustments to the probabilistic performance feedback and therefore allowed testing the stability of learning in the absence of feedback. Note, however, that in contrast to Peterburs et al. (2022), we included all trials, i.e., both from training and test blocks, in the statistical analyses.

*Presentation software* (version 20.1, Neurobehavioral Systems, Inc., Albany, CA, USA) was used for stimulus presentation and response recording. Participants received either course credit or monetary compensation for taking part in this study. Task completion took around 35 min.

### Stimulus selection

Cue stimuli consisted of colored images of adult faces and were obtained from the FACES database (Ebner et al., 2010; https://faces.mpdl.mpg.de/imeji/). We selected four pairs of images of individuals. For each pair, one image shows an emotional (positive, i.e. happy, or negative, i.e. angry) and one image a non-emotional (neutral) face expression, resulting in two sets of four facial stimuli each, one emotional set and one neutral set. Importantly, both stimulus sets contained images of the same individuals, differing only in face emotionality, i.e., emotional vs. neutral. The emotional stimulus set contained images of four young adults (of similar age as our study participants), two males and two females: two (one male and one female) with a happy and two (one male and one female) with an angry facial expression. The non-emotional stimulus set contained images of the same young adults, all four with a neutral facial expression. Facial stimuli were selected based on ratings of an independent sample of 154 adults on perceived facial expression, perceived age, attractiveness, and distinctiveness provided by Ebner et al. (2010) and Ebner et al. (2018), with the selected images rated as similar as possible according to those variables. Stimulus assignment to the experimental conditions within each stimulus set was randomized. For illustrative purposes, exemplary facial cues that are publicly available via the FACES platform are presented in Fig. 2. Additional information about the selected stimuli including filenames as well as stimulus ratings are provided in the Supplementary Material.

### Data analysis

Before analyzing the data, we performed three quality control tests to prevent including participants with low compliance and/or motivation. First, participants with more than 25% invalid button presses, that is button presses that were too slow or indicated the opposite location of the target stimulus (open circle), were excluded in accordance with Scholz et al. (2022). Second, we excluded participants choosing the same action option (*go* or *nogo*) in more than 85% of trials as this indicated failure to understand the task instructions of using both response options properly, similar to Weber et al. (2022). Finally, we excluded participants with choice accuracy below-chance level (according to a one-tailed binomial test against chance, α = .05) in GW trials, i.e., the easiest condition (see Weber et al., 2022), as previous findings indicated a ceiling effect for GW learning (e.g. Peterburs et al., 2022; Peterburs et al., 2021). Based on those criteria, data from 15 participants were excluded from further analyses: 12 participants showed perseverative responding for either go or nogo, five participants’ performance was below chance level in the easiest condition (GW), and one participant had incomplete data due to technical problems during data acquisition. Beyond that, we removed invalid trials, i.e., button presses outside the RT window (> 1000 ms) or button presses indicating the opposite location of the target stimulus (open circle) prior to data analysis. By applying these criteria, 3.51% of trials were removed.

We performed a linear mixed-effects (LME) model analysis on aggregated choice accuracy data using the *lme4* package in R (Bates et al., 2014). As categorical fixed-effect predictors, we included the between-subjects factor learning condition (*neutral* [= reference level], *congruent*, *incongruent*) as well as the within-subject factors action (*go* [= reference level], *nogo*), outcome valence (*win* [= reference level], *avoid-losing*), and block type (*training* [= reference level], *test*). Contrast weights of categorical predictors were set using deviation coding. Block (1–8) was implemented as a continuous predictor to assess effects of task duration and was centered and scaled to the grand mean. Aggregated accuracy in percent was set as the dependent variable. Pavlovian bias was quantified as the effect of a two-way interaction between action and outcome valence.

We used the *bobyqa* algorithm for parameter optimization. The maximal number of iterations was set to 10e6. LME models were fitted using a restricted maximum likelihood approach, as proposed by Luke (2017). Degrees of freedom and *p*-values were calculated using the likelihood ratio test method that was based on type III sums of squares. The *lmerTest* package (Kuznetsova et al., 2017) was used to calculate *p*-values based on Satterthwaite’s approximation of degrees of freedom. We attempted to incorporate maximal random-effects structure with all within-subjects effects and interactions as random slopes and random intercepts per participant (Barr, 2013). Since the full model with a maximal random-effects structure resulted in a singular fit, we simplified the model manually via stepwise elimination of random effects. The simplified LME model for choice accuracy data that still reached convergence was specified as follows:

$$\eqalign{ choice\,accuracy \sim & \,learning\,condition\,*\,action\,*\,valence\,* \cr & blocktype\,*\,block\, + \,(1\, + \,action\,*\,valence\,* \cr & block\, + \,action:\,block\,type\,|\,participant) \cr}$$

In order to identify statistical outliers, we calculated Cook’s distance using the influence.ME package. In case participants exceeded the Cook’s distance cut-off criterion of 4/(*n* - *k* − 1), with *n* = number of participants and *k* = number of fixed effects including the intercept as well as all main and interaction effects, they were excluded and the LME model was refitted for the remaining participants. Cook’s distance indicated that one participant from INC had to be excluded. Refitting the LME model specified above for the remaining 121 participants (see Table 1 for sample characteristics after exclusions) resulted in a singular fit. We therefore further simplified the random effects structure until the model converged. The final LME model for choice accuracy data was specified as follows:

$$\eqalign{ choice\,accuracy \sim & \,learning\,condition\,*\,action\,*\,valence\,* \cr & block\,type\,*\,block\, + \,(1\, + \,action\,*\,valence\,* \cr & block\, + \,block\,type\,|\,participant) \cr}$$

Pairwise comparisons for the three-level factor learning condition as well as significant interaction effects were resolved via the R package *emmeans* (Lenth, 2022). To elucidate three-way or higher-order interactions involving the interaction effect between action and valence, in which all involved factors were categorical, we additionally explored *Pavlovian congruency gain indexes*, similar to Wagenbreth et al. (2015). Note that in this context the term congruency denotes what we refer to as Pavlovian conflict. Following that *Pavlovian congruency gains* reflect the gain in accuracy for win compared to avoid-losing trials as calculated by subtracting accuracies of avoid-losing from win conditions. This value is typically positive for go trials (because GW > GAL) and negative for nogo trials (because NGAL > NGW). Note that, except for interaction effects involving the predictors action and valence, we will only report significant interaction effects of factors which are not included in higher-order interactions. For all reported statistical analyses, the threshold for statistical significance was set to *p* = .05. For follow-up comparisons, adjusted *p*-values using false-discovery-rate (FDR) correction for multiple comparisons are reported (Benjamini & Hochberg, 1995). Note that we report unstandardized effect size estimates by providing β coefficients for each statistical test. In addition, we report complementary standardized effect size estimates for significant main and interaction effects using partial eta squared (*η*_*p*_^*2*^) implemented in the *effectsize* package in R (Ben-Shachar et al., 2020). Model specification and results for the analyses of RTs and rating data are reported in the Supplementary Material.

Finally, we assessed whether reward and punishment sensitivity as measured using the BIS/BAS scales moderated the Pavlovian bias in an exploratory analysis. To this end, we calculated Spearman rank-order correlations between participant-wise random slopes for the action × valence interaction effect and BIS and BAS scores, similar to Weber et al. (2022).

## Results

### Linear mixed-effects model analysis of choice accuracy data

#### General effects on learning performance

The inferential statistics for all fixed effects of the LME model analysis are listed in Table 2. LME model analysis of choice accuracy revealed significant main effects of block, *F*(1, 118) = 39.03, *p* < .001, *η*_*p*_^*2*^ = 0.25 (95%-CI 0.13–0.37), and block type, *F*(1, 121.05) = 7.40, *p* = .007, *η*_*p*_^*2*^ = 0.06 (95%-CI 0-0.15), indicating that accuracy linearly increased throughout the task, β = 3.13 (*SE* = 0.50), and that overall performance was better in test compared to training blocks, β = 1.70 (*SE* = 0.62). Furthermore, results yielded a significant main effect of action, *F*(1, 118.98) = 120.40, *p* < .001, *η*_*p*_^*2*^ = 0.50 (95%-CI 0.38–0.60), with better overall performance in go relative to nogo conditions, β = − 20.22 (*SE* = 1.84). Importantly, there was a significant main effect of learning condition, *F*(2, 118.72) = 4.38, *p* = .015, *η*_*p*_^*2*^ = 0.07 (95%-CI 0-0.16). Pairwise comparisons indicated better learning performance in CON relative to INC, β = 7.81 (*SE* = 2.64), *p* = .011, FDR-corrected. There were no significant differences in overall learning performance between CON and NEU, *p* = .153, FDR-corrected, or INC and NEU, *p* = .153, FDR-corrected. Results for the analysis of RTs and rating data are reported in the Supplementary Material.

**Table 2:** Inferential statistics for the fixed effects of the linear-mixed effects model of choice accuracy

Fixed effect	β	SE	df	*F* /* t*	*p*
(intercept)	65.90	1.07	118.72	61.45	< .001***
**learning condition**			**2, 118.72**	**4.38**	.015*
*NEU vs. CON*	*4.04*	*2.62*	*118.72*	*1.54*	*.126*
*NEU vs. INC*	*-3.77*	*2.62*	*118.72*	*-1.44*	*.153*
**action**	**-20.22**	**1.84**	**1, 118.98**	**120.40**	< .001***
valence	2.48	1.59	1, 119.33	2.43	.121
**block type**	**1.70**	**0.62**	**1, 121.05**	**7.40**	.007**
**block**	**3.13**	**0.50**	**1, 118**	**39.03**	< .001***
learning condition:action			2, 118.98	1.81	.167
*NEU vs. CON*	*8.44*	*4.51*	*118.98*	*1.87*	*.063*
*NEU vs. INC*	*2.83*	*4.51*	*118.98*	*0.63*	*.531*
learning condition:valence			2, 119.33	2.08	.130
***NEU vs. CON***	***-7.89***	***3.88***	***119.33***	***-2.03***	.044*
*NEU vs. INC*	*-4.44*	*3.88*	*119.33*	*-1.14*	*.255*
**action:valence**	**35.37**	**3.68**	**1, 118.99**	**92.59**	< .001***
learning condition:block type			2, 121.05	0.27	.760
*NEU vs. CON*	*1.12*	*1.52*	*121.05*	*0.74*	*.463*
*NEU vs. INC*	*0.67*	*1.52*	*121.05*	*0.44*	*.661*
**action:block type**	**8.25**	**1.09**	**1, 2767.67**	**57.72**	< .001***
valence:block type	-0.75	1.09	1, 2767.67	0.48	.488
learning condition:block			2, 118	< 0.01	1.000
*NEU vs. CON*	*-0.01*	*1.22*	*118.00*	*-0.01*	*.993*
*NEU vs. INC*	*-0.01*	*1.22*	*118.00*	*-0.01*	*.994*
**action:block**	**2.74**	**1.28**	**1, 120.05**	**4.57**	.034*
valence:block	1.16	0.77	1, 123.78	2.28	.133
**block type:block**	**-1.42**	**0.54**	**1, 2767.67**	**6.86**	.009**
**learning condition:action:valence**			**2, 118.99**	**7.28**	.001**
***NEU vs. CON***	***-28.67***	***8.98***	***118.99***	***-3.19***	.002**
***NEU vs. INC***	***-30.51***	***8.98***	***118.99***	***-3.40***	.001**
learning condition:action:block type			2, 2767.67	1.75	.173
*NEU vs. CON*	*-3.58*	*2.66*	*2767.67*	*-1.35*	*.178*
*NEU vs. INC*	*1.24*	*2.66*	*2767.67*	*0.47*	*.641*
learning condition:valence:block type			2, 2767.67	0.04	.961
*NEU vs. CON*	*0.39*	*2.66*	*2767.67*	*0.15*	*.885*
*NEU vs. INC*	*0.75*	*2.66*	*2767.67*	*0.28*	*.779*
**action:valence:block type**	**12.43**	**2.17**	**1, 2767.67**	**32.74**	< .001***
learning condition:action:block			2, 120.05	0.50	.605
*NEU vs. CON*	*2.91*	*3.13*	*120.05*	*0.93*	*.354*
*NEU vs. INC*	*0.41*	*3.13*	*120.05*	*0.13*	*.897*
learning condition:valence:block			2, 123.78	1.76	.176
*NEU vs. CON*	*-2.58*	*1.88*	*123.78*	*-1.37*	*.173*
*NEU vs. INC*	*-3.38*	*1.88*	*123.78*	*-1.79*	*.076*
**action:valence:block**	**4.08**	**1.87**	**1, 121.89**	**4.77**	.031*
learning condition:block type:block			2, 2767.67	0.40	.671
*NEU vs. CON*	*-1.13*	*1.33*	*2767.67*	*-0.85*	*.396*
*NEU vs. INC*	*-0.24*	*1.33*	*2767.67*	*-0.18*	*.859*
**action:block type:block**	**-4.99**	**1.09**	**1, 2767.67**	**21.12**	< .001***
valence:block type:block	-0.49	1.09	1, 2767.67	0.21	.650
learning condition:action:valence:block type			2, 2767.67	0.85	.428
*NEU vs. CON*	*-6.86*	*5.31*	*2767.67*	*-1.29*	*.197*
*NEU vs. INC*	*-4.21*	*5.31*	*2767.67*	*-0.79*	*.429*
learning condition:action:valence:block			2, 121.89	0.31	.731
*NEU vs. CON*	*0.07*	*4.57*	*121.89*	*0.02*	*.988*
*NEU vs. INC*	*3.18*	*4.57*	*121.89*	*0.70*	*.487*
learning condition:action:block type:block			2, 2767.67	0.52	.593
*NEU vs. CON*	*-0.37*	*2.66*	*2767.67*	*-0.14*	*.890*
*NEU vs. INC*	*2.16*	*2.66*	*2767.67*	*0.81*	*.417*
learning condition:valence:block type:block			2, 2767.67	0.65	.524
*NEU vs. CON*	*-2.61*	*2.66*	*2767.67*	*-0.98*	*.327*
*NEU vs. INC*	*-2.62*	*2.66*	*2767.67*	*-0.99*	*.324*
action:valence:block type:block	-4.01	2.17	1, 2767.67	3.41	.065
learning condition:action:valence:block type:block			2, 2767.67	0.76	.469
*NEU vs. CON*	*4.36*	*5.31*	*2767.67*	*0.82*	*.412*
*NEU vs. INC*	*6.39*	*5.31*	*2767.67*	*1.20*	*.229*

*Note.* SE = standard error, df = degrees of freedom; the *t*-statistic rather than the *F*-statistic is provided for contrasts related to the three-level predictor learning condition (corresponding rows are italicized); statistically significant results are highlighted in bold

*p < .05, **p < .01, ***p < .001

#### Modulation of Pavlovian biases by emotional cues

**Fig. 3:**
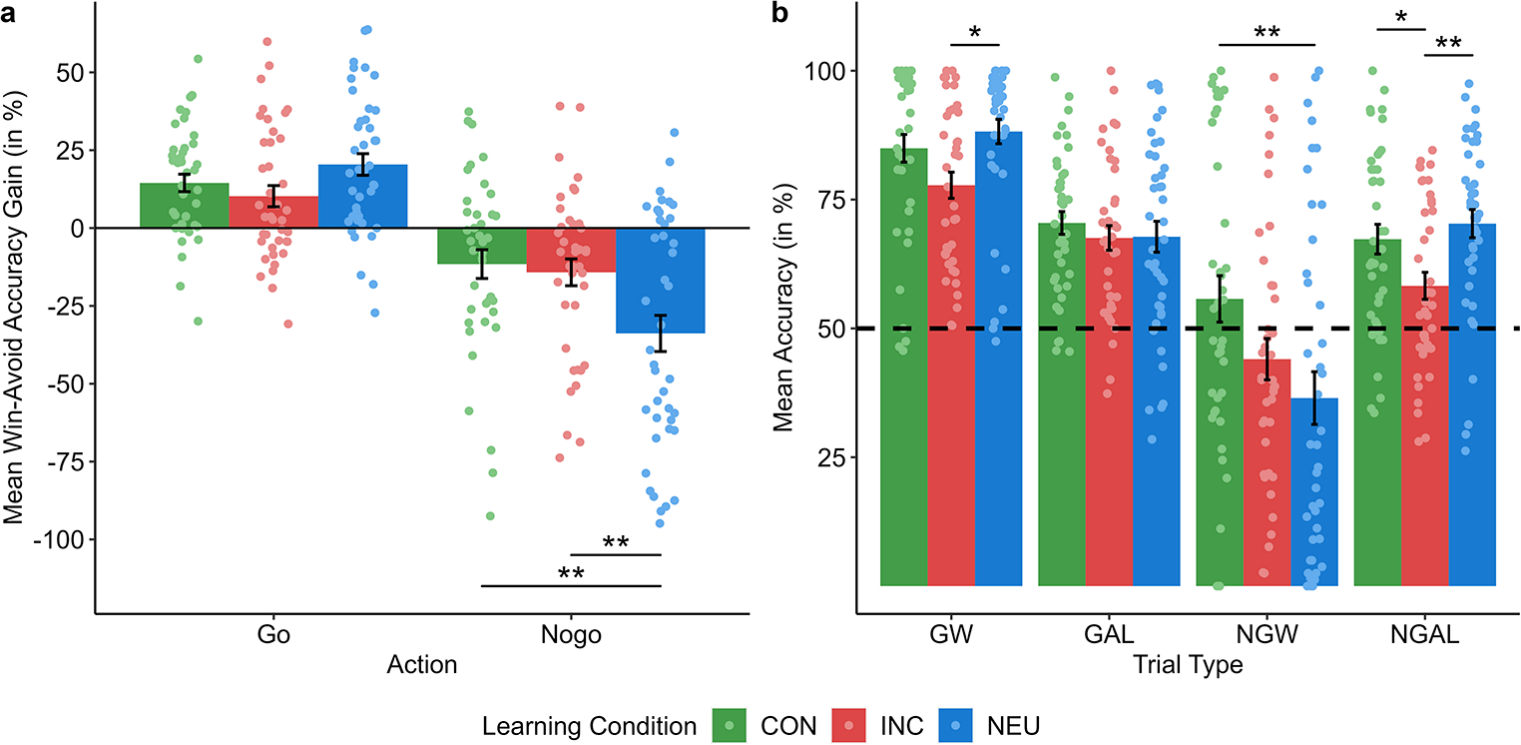
Pavlovian bias modulated by learning condition. (**a**) Mean Pavlovian congruency gain indexes for go (left) and nogo trials (right) according to learning condition (green bars = emotional-congruent learning condition, red bars = emotional-incongruent learning condition, blue bars = neutral learning conditions). Scores represent the interaction between action and valence in choice accuracy (difference of win and avoid-losing). The lighter shaded dots represent subject-level choice accuracies. Error bars are represented as the standard error of the mean. (**b**) Mean accuracy across all trials is plotted for each experimental condition (GW = go to win, GAL = go to avoid losing, NGW = nogo to win, NGAL = nogo to avoid losing), according to learning condition (green bars = emotional-congruent learning condition, red bars = emotional-incongruent learning condition, blue bars = neutral learning conditions). The lighter shaded dots represent subject-level choice accuracies. Error bars are represented as the standard error of the mean. *p < .05, **p < .01, ***p < .001

Crucially, we found an action × valence interaction effect, reflecting the Pavlovian bias, *F*(1, 118.99) = 92.59, *p* < .001, *η*_*p*_^*2*^ = 0.44 (95%-CI 0.31–0.54). Performance was better for GW relative to GAL, β = -15.21 (*SE* = 1.88), *t*(119.90) = -8.09, *p* < .001, FDR-corrected, and for NGAL relative to NGW, β = 20.16 (SE = 2.87), *t*(118.81) = 7.01, *p* < .001, FDR-corrected. Importantly, the observed Pavlovian bias effect was modulated by the factor learning condition as reflected in a significant learning condition × action × valence interaction effect, *F*(2, 118.99) = 7.28, *p* = .001, *η*_*p*_^*2*^ = 0.11 (95%-CI 0.02–0.22) (see Fig. 3).

To resolve this interaction, we first checked whether the action × valence interaction was evident in each learning condition. Results revealed that the action × valence interaction effect emerged under all three learning conditions (all *p*s < 0.001, FDR-corrected, all *η*_*p*_^*2*^ ≥ 0.11). Notably, the interaction coefficient was significantly higher for NEU, β = 55.09 (*SE* = 6.31), relative to both CON, β = 26.42 (*SE* = 6.39), *t*(118.99) = 3.19, *p* = .003, FDR-corrected, and INC, β = 24.58 (*SE* = 6.39), *t*(118.99) = 3.40, *p* = .003, FDR-corrected. Interaction coefficients did not differ between CON and INC, *p* = .840, FDR-corrected.

To further characterize the effect of learning condition on the action × outcome interaction effect, we next compared *Pavlovian congruency gain indexes*, quantified as the gain in accuracy for win minus avoid-losing, separately for go and nogo between learning conditions (see Fig. 3a). For go, gain indexes did not differ between learning conditions, all *ps* ≥ 0.061, FDR-corrected. However, for nogo, absolute values for the (negative) gain indexes were reduced for CON relative to NEU, Δ_win−avoid_ = -22.22 (*SE* = 7.03), *t*(118.81) = -3.16, *p* = .006, FDR-corrected, and for INC relative to NEU, Δ_win−avoid_ = -19.69 (*SE* = 7.03), *t*(118.81) = -2.90, *p* = .009, FDR-corrected. There was no difference between CON and INC, *p* = .721, FDR-corrected.

As a last step, we explored what caused these differences among learning conditions by comparing learning performance for each action-valence combination between learning conditions (see Fig. 3b). For GW, INC showed significantly decreased learning performance compared to NEU, β = -10.59 (*SE* = 3.69), *t*(119.56) = 2.95, *p* = .011, FDR-corrected, while there was no difference in learning performance between INC and CON, *p* = .073, FDR-corrected, and NEU and CON, *p* = .343, FDR-corrected. Similarly, for NGAL, learning performance was significantly decreased in INC relative to NEU, β = -12.20 (*SE* = 3.89), *t*(119.32) = 3.14, *p* = .007, FDR-corrected, and CON, β = 9.35 (*SE* = 3.92), *t*(119.32) = 2.39, *p* = .028, FDR-corrected. However, differences in learning performance between NEU and CON were again non-significant, *p* = .465, FDR-corrected. Contrary to that, learning performance did not differ between learning conditions for GAL (all *p*s ≥ 0.664, FDR-corrected). Finally, results indicated improved NGW learning performance for CON compared to NEU, β = 19.37 (*SE* = 6.47), *t*(118.48) = -3.00, *p* = .010, FDR-corrected, but no differences between CON and INC (*p* = .106, FDR-corrected) or NEU and INC (*p* = .249, FDR-corrected).

In summary, the Pavlovian bias effect emerged under all learning conditions but was reduced in both CON and INC relative to NEU, particularly for nogo-learning. Crucially, while the reduced Pavlovian bias found in CON was driven by improved learning performance in NGW, the reduced Pavlovian bias effect in INC was rooted in impaired learning performance in GW and NGAL.

#### Complementary results

##### Increased Pavlovian biases in the absence of feedback

**Fig. 4:**
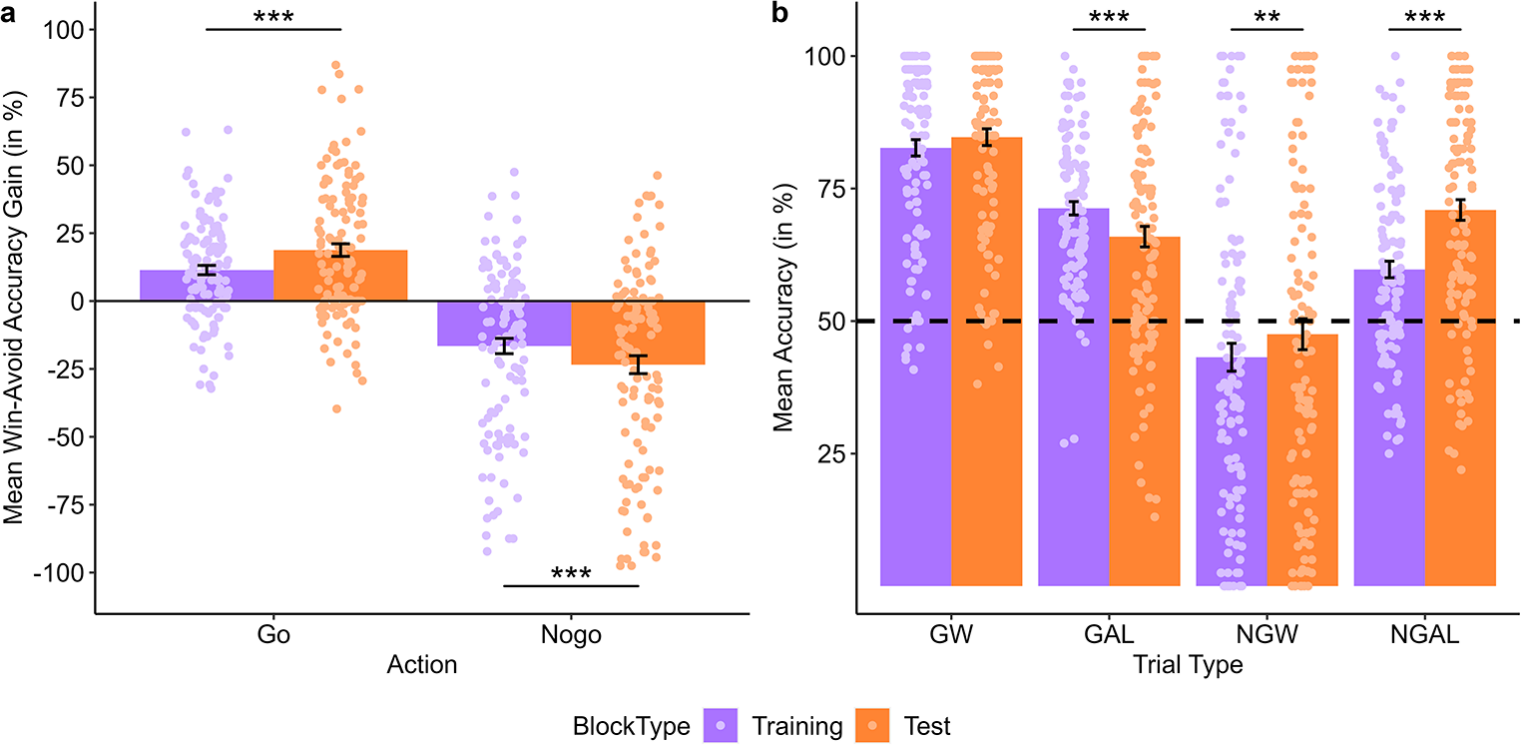
Pavlovian bias modulated by block type. (**a**) Mean Pavlovian congruency gain indexes for go (left) and nogo trials (right) according to block type (purple bars = training blocks, orange bars = test blocks). Scores represent the interaction between action and valence in choice accuracy (difference of win and avoid-losing). The lighter shaded dots represent subject-level choice accuracies. Error bars are represented as the standard error of the mean. Error bars are represented as the standard error of the mean. (**b**) Mean accuracy across all trials is plotted for each experimental condition (GW = go to win, GAL = go to avoid losing, NGW = nogo to win, NGAL = nogo to avoid losing), according to block type (purple bars = training blocks, orange bars = test blocks). The lighter shaded dots represent subject-level choice accuracies. Error bars are represented as the standard error of the mean. Error bars are represented as the standard error of the mean. *p < .05, **p < .01, ***p < .001

Beyond that, results revealed an action × valence × block type interaction effect, *F*(1, 2767.67) = 32.74, *p* < .001, *η*_*p*_^*2*^ = 0.01 (95%-CI 0.01–0.02; see Fig. 4).The action × valence interaction effect was significant for both block types (both ps < .001, FDR-corrected, both *η*_*p*_^*2*^ ≥ 0.29). However, the interaction coefficient was larger for test, β = 41.58 (*SE* = 3.83, compared with training blocks, β = 29.15 (*SE* = 3.83), *t*(2767.67) = 5.72, *p* < .001. Comparing Pavlovian congruency gain indexes separately for go and nogo between block types revealed increased (absolute) gain indexes in test relative to training blocks for both go, Δ_win−avoid_ = 6.97 (SE = 1.54), *t*(2767.67) = 4.54, *p* < .001, and nogo conditions, Δ_win−avoid_ = -5.46 (*SE* = 1.54), *t*(2767.67) = -3.56, *p* < .001 (see Fig. 4a). Comparing block types at each action-valence combination further indicated increased learning performance in test relative to training blocks in NGAL, β = 8.56 (*SE* = 1.13), *t*(1052.62) = 7.58, *p* < .001, FDR-corrected, and NGW, β = 3.09 (SE = 1.13), *t*(1052.62) = 2.74, *p* = .008, FDR-corrected. In contrast, GAL learning performance was increased in training relative to test blocks, β = -5.92 (*SE* = 1.13), *t*(1052.62) = -5.24, *p* < .001, FDR-corrected (see Fig. 4b). There was no difference between block types for GW, *p* = .351, FDR-corrected.

In summary, the Pavlovian bias was increased, in test blocks, i.e. in the absence of feedback for both go and nogo learning. Importantly, accuracy was decreased in GAL, but increased in NGAL and NGW in test relative to training blocks.

##### Effects of the continuous factor block on learning performance

Furthermore, LME model analysis revealed an action × valence × block interaction effect, *F*(1, 121.89) = 4.77, *p* = .031, *η*_*p*_^*2*^ = 0.04 (95%-CI 0-0.12). Slope estimates for the block factor were significant for GW, NGW and NGAL, all *p*s ≤ 0.033, FDR-corrected, but not GAL, *p* = .149, FDR-corrected, indicating accuracy increases across blocks in all learning conditions, βs ≥ 2.20 (*SE*s ≤ 0.1.28), except GAL. Subsequent comparisons of slope estimates for each action-valence combination revealed that the increase in accuracy across blocks was strongest in NGAL, β = 6.10 (*SE* = 0.97), thus greater relative to NGW, β = 2.90 (*SE* = 1.28), *t*(121.75) = -2.38, *p* = .038, FDR-corrected, GAL, β = 1.32 (*SE* = 0.91), *t*(121.19) = -3.29, *p* = .008, FDR-corrected, and GW, β = 2.20 (*SE* = 0.85), *t*(121.94) = -2.97, *p* = .011, FDR-corrected. All other comparisons did not reach statistical significance (all *p*s ≥ 0.491, FDR-corrected).

There was also an action × block type × block interaction effect, *F*(1, 2767.67) = 21.12, *p* <. 001, *η*_*p*_^*2*^ = 0.01 (95%-CI 0-0.02). For go conditions, slope estimates for the predictor block indicated accuracy increases across test, β = 2.30 (*SE* = 0.80), *p* = .009, FDR-corrected, but not training blocks, *p* = .129, FDR-corrected, though slope estimates did not significantly differ from each other, *t*(2767.67) = 1.40, *p* = .162. For nogo conditions, slope estimates for the predictor block indicated accuracy increases across both training, β = 6.46 (*SE* = 0.99), *p* < .001, FDR-corrected, and test blocks, β = 2.54 (*SE* = 0.99), *p* = .011, FDR-corrected, with greater increases over blocks for training relative to test blocks, *t*(2767.67) = -5.10, *p* < .001.

In summary, choice accuracy linearly increased throughout the task for all experimental conditions, except for GAL, with the largest increase in accuracy across blocks for NGAL. Crucially, increases in learning performance for go conditions were only observed in test but not training blocks, while for nogo conditions accuracy increased across both training and test blocks.

### Relationship between BIS/BAS and the Pavlovian bias

Relationships between reward and punishment sensitivity and the Pavlovian bias were explored via Spearman rank-order correlations between participants’ BIS/BAS scores and participant-wise random slopes for the action × valence interaction effect. However, results did not reveal significant correlations, r(119) ≤ |0.046|, all ps ≥ 0.920, FDR-corrected. Importantly, calculating correlations separately for each learning condition also failed to reveal a significant relationship between BIS/BAS scores and random slopes for the action × valence interaction, CON: r(38) ≤ |0.159|, all ps ≥ 0.490, FDR-corrected, INC: r(38) ≤ |0.349|, all ps ≥ 0.082, FDR-corrected, NEU: r(39) ≤ |0.242|, all ps ≥ 0.380, FDR-corrected.

## Discussion

In the present study, we aimed to test whether and how the Pavlovian bias in feedback-based learning can be modulated by social affective cues. Participants were tested using a variant of the orthogonalized Go/Nogo task which fully decoupled action and outcome valence. Importantly, participants learned action-outcome contingencies either from neutral (NEU) or emotional facial cues, for which action tendencies associated with the affective valence of the facial cue were either congruent (CON) or incongruent (INC) to the required action in order to obtain the best possible outcome on each trial. We hypothesized that when action tendencies associated with the affective valence of the facial cue matched the required instrumental action, the Pavlovian bias would be reduced relative to learning from neutral cues. Conversely, when action tendencies associated with the affective valence of the facial cue contradicted the required instrumental action, we expected the Pavlovian bias to be enhanced relative to learning from neutral cues (although this hypothesis was somewhat more speculative, see above). We assumed emotional faces to primarily affect learning performance in conflict conditions (i.e. GAL and NGW). The results revealed that the Pavlovian bias was present ubiquitously in all three learning conditions. However, contrary to our hypotheses, the Pavlovian bias was reduced in both CON and INC relative to NEU.

As expected, participants learned better to execute a response to obtain a reward than to avoid punishment, and to withhold a response to avoid punishment than to obtain a reward. In line with our hypotheses, this asymmetry observed in instrumental learning, referred to as Pavlovian bias, was evident in each learning condition. This result is largely consistent with previous research reporting a robust Pavlovian bias in various study populations and task contexts (e.g. Cavanagh et al., 2013; Dorfman & Gershman, 2019; Ereira et al., 2021; Guitart-Masip et al., 2012, 2014a; Peterburs et al., 2021, 2022; Raab & Hartley, 2020; Scholz et al., 2022). The Pavlovian bias can be understood as a conflict between hard-wired *Pavlovian control of behavior*, in which the anticipation of valenced outcomes automatically determines the choice of action (i.e., anticipating reward facilitates action invigoration, and anticipating punishment facilitates action inhibition), and flexible *instrumental control of behavior* which slowly but efficiently adjusts our actions to obtain the best possible outcome. At the neural level, this might be rooted in the ventral striatal dopamine (DA) system, signaling reward prediction errors (Bayer & Glimcher, 2005; Schultz et al., 1997). Accordingly, better-than-expected outcomes elicit increased striatal DA release that facilitates action invigoration, and worse-than-expected outcomes elicit reduced striatal DA release which facilitates action inhibition (Collins & Frank, 2014; Frank et al., 2004; Schultz et al., 1997; Wickens et al., 2007). Accordingly, cues predicting reward would lead to increased firing of DA neurons, thus facilitating *go* responses, and cues predicting punishment would lead to reduced firing of DA neurons, thus facilitating *nogo* responses. Note however, that a role of prefrontal dopamine in the resolution of Pavlovian-instrumental conflicts has also been considered (Scholz et al., 2022), which is in line with previous findings that linked the prefrontal cortex to overcoming Pavlovian biases (Cavanagh et al., 2013; Kim et al., 2023).

Notably, in the present study, the Pavlovian bias was attenuated in CON. This effect was driven by a selective performance boost in the conflict condition NGW that was cued by an angry face. While this result is consistent with our hypotheses, against our expectations the manipulation did not influence learning performance in GAL. Previous research has consistently linked appetitive and aversive stimuli, including facial expressions, with approach and avoidance motivation, respectively (Marsh et al., 2005; Nikitin & Freund, 2019; Phaf et al., 2014; Seidel et al., 2010). Our results demonstrate that during reinforcement learning, at least in the context of nogo learning, maladaptive Pavlovian biases can be reduced by learning from angry facial cues. Findings from Chiu et al. (2014) further suggest that this effect may originate in the motor system, as they could show that aversive cues decreased motor system excitability, therefore biasing nogo behavior in a Go/Nogo task. Beyond that, overcoming Pavlovian biases has been linked to effort-based resolution of motivational conflicts via cognitive control mechanisms (Cavanagh et al., 2013). Emotional valence has been shown to be capable of resolving both emotional and cognitive conflicts (Kanske & Kotz, 2010, 2011; Zinchenko et al., 2015, 2020). Thus, angry faces may have helped resolve the Pavlovian conflict related to NGW. Importantly, the modulatory effect of CON was restricted to nogo, highlighting the robustness of the Pavlovian learning bias in the context of go, consistent with previous studies (e.g. Asci et al., 2019; Ereira et al., 2021; Peterburs et al., 2021; Weber et al., 2022).

Crucially, the Pavlovian bias was not only reduced in CON but also in INC. However, what caused the observed result patterns for CON and INC was fundamentally different. For CON, affective cues supported regulating maladaptive Pavlovian influences by improving performance in the conflict condition NGW, thereby promoting adaptive and goal-directed decision-making (at least in the nogo context). In contrast, for INC, affective cues did not affect learning performance in conflict conditions but rather led to impaired performance in both non-conflict conditions, GW and NGAL, which in turn resulted in a reduced Pavlovian nogo bias. Even though not in line with our hypotheses, it should be noted that this results still confirms the assumed relationship between affective cues and approach-avoidance behavior, with angry faces impairing learning performance in GW and happy faces impairing learning performance in NGAL. In addition, although both CON and INC were associated with comparably reduced Pavlovian biases, global learning success was reduced in INC relative to CON. These findings suggest that reduced Pavlovian biases and adaptive, goal-directed behavior reflected in the global learning performance do not always go hand in hand and should therefore be dissociated.

Taking that into consideration, improved Pavlovian-instrumental regulation observed in both CON and INC relative to NEU was only adaptive in CON but not INC, as only for CON an improvement in instrumental performance in one of the conflict conditions could be detected. Thus, although seemingly similar at first glance, our results indicate opposite effects of CON and INC related to goal-directed behavior.

Strikingly, non-conflict and conflict conditions have been shown to be differently sensitive to emotionally valenced cues in CON versus INC. Why affective cues modulated conflict in CON but non-conflict conditions in INC still remains speculative. One possible reason may be congruency differences between CON and INC related to outcome valence. Thompson and Westwater (2017) demonstrated that happy, neutral, and angry faces can be successfully used as social feedback signalizing win, draw, and loss, respectively. In the present study, however, facial stimuli were implemented as learning cues instead of feedback stimuli. Importantly, congruency was based on whether the valence of the facial cue was in accordance with the required response, thus action-congruent (e.g. happy face for go conditions, i.e. GW and GAL) but not outcome-congruent (which would have used happy faces for win conditions, i.e. GW and NGW). Poorer learning performance for INC in non-conflict conditions GW and NGAL might therefore be explained by the fact that both GW and NGAL were not only action- but also outcome-incongruent, thus further cognitive control would have been needed to resolve two levels of maladaptive conflicts, i.e. both action-incongruencies as well as outcome-incongruencies in those conditions. However, this interpretation only holds for INC. For CON, both non-conflict conditions were not only action- but also outcome-congruent. Thus, according to this interpretation, one would assume task performance to even increase in GW and NGAL due to two levels of facilitation. While it might be plausible to not find an additional increase in performance in the easiest condition GW, due to a ceiling effect (e.g. Peterburs et al., 2021), our results also did not provide evidence for improved task performance in NGAL.

Lerner et al. (2015) emphasized that emotional valence constitutes only one of multiple dimensions related to emotional processing. A reduced Pavlovian bias for both CON and INC might therefore be better explained by increased arousal in response to the cue and/or increased cue salience which may improve Pavlovian regulation. However, as we have discussed, improved Pavlovian-instrumental regulation seems not always to be adaptive. Thus, cue valence, or cue-action congruency may determine whether the reduction in Pavlovian bias is adaptive or maladaptive with respect to goal-directed behavior. Our results therefore expand previous studies that investigated affective valence as the effect of emotional content (Asci et al., 2019) and induced affect (Weber et al., 2022) on the Pavlovian bias but failed to unveil modulatory effects, potentially as those manipulations did not affect cue salience. Our results suggest that, in order to modulate Pavlovian biases, manipulating both cue valence and stimulus salience may be inevitable, suggesting that modulation of the Pavlovian bias effect may require affective evaluation (see also Eder & Rothermund, 2008; Rotteveel & Phaf, 2004). However, it has to be noted that a potential role of arousal/salience in resolving Pavlovian learning biases is rather speculative, as arousal/salience was neither directly manipulated or rated in the present study nor were arousal ratings collected in the validation study by Ebner et al. (2010).

Previous studies indicated Pavlovian biases to be robust to manipulations (e.g. Asci et al., 2019; Ereira et al., 2021; Peterburs et al., 2021; Weber et al., 2022). Nevertheless, Ereira et al. (2021) could show reduced Pavlovian biases through training in a task variant, that used a semantic response domain, combined with gamification and spaced stimulus presentation. Notably, these results may also be explained by increased arousal/salience related to gamification and the semantic domain that hint at a potential role of arousal/salience in overcoming Pavlovian biases. Crucially, emotional faces do not only differ from neutral faces with respect to arousal and salience, but also in terms of motivational intensity (Harmon-Jones et al., 2012a, 2012b, 2013), i.e. the strength of the urge to approach or avoid. However, note that motivational intensity was found to be closely related to valence (Campbell et al., 2021). Beyond that, emotional faces also seem to differ from neutral faces in terms of the appraisal of affective relevance, which has been shown to underly learning biases in Pavlovian aversive conditioning (Stussi et al., 2018, 2021). Thus, it remains unclear which specific mechanism underlies the observed modulation of the Pavlovian bias, reported in the present study. Additional research with direct manipulation of cue arousal/salience and related concepts including motivational intensity and affective relevance is needed to clarify and further characterize potential modulators of the Pavlovian bias on instrumental learning and to evaluate its clinical potential.

Still, to our knowledge, our results provide first evidence for an easy-to-implement intervention, without the need of additional training, that is capable of diminishing Pavlovian-instrumental interference, reflected in reduced Pavlovian bias on instrumental learning.

Interestingly, Pavlovian bias proved to be higher in test blocks in which participants were not provided feedback for their choices compared with training blocks which contained trial-by-trial feedback. This finding fits well with the theory of Pavlovian-instrumental arbitration, proposed by Dorfman and Gershman (2019). According to this theory, the brain arbitrates between Pavlovian and instrumental control of behavior depending on which can better predict reward. While under Pavlovian control, reward predictions are generated based on stimuli only, reward predictions under instrumental control not only consider stimuli but also actions. Dorfman and Gershman (2019) theorized that if actions do not or unreliably affect consequences and rewards are hence uncontrollable, the brain favors Pavlovian over instrumental control, resulting in increased Pavlovian bias. Indeed, Dorfman and Gershman (2019) found that Pavlovian bias was stronger under conditions of low relative to high reward controllability by manipulating the probability of rewards in a reward-based Go/Nogo task. The arbitration theory has been also linked to learned helplessness, a hypothesized model to underly anxiety and depressive symptoms (Csifcsák et al., 2020; Dorfman & Gershman, 2019; Mineka & Hendersen, 1985).

Completely omitting performance feedback might constitute a similar state of reduced reward controllability. During training blocks, rewards could be directly controlled through one’s actions, thus instrumental control was useful to flexibly adjust the current response strategy depending on the previous feedback received. In test blocks, when feedback was omitted, feedback-guided decision-making was not possible anymore, and rewards might have seemed uncontrollable. Note however, that participants were instructed that correct decisions were also rewarded in test blocks. Our finding of increased Pavlovian bias in test blocks compared with training blocks may therefore be explained in terms of stronger reliance on a Pavlovian predictor for rewards under conditions of lower reward controllability (i.e., when actions did not have immediate consequences).

A recent study by Kurtenbach et al. (2022) directly compared task performance during reinforced and non-reinforced periods in a Go/Nogo task. Results indicated a shift in response strategy to a more cautious response style with a decreased tendency to execute a response in non-reinforced relative to reinforced trials that was accompanied by a general improvement in non-reinforced relative to reinforced trials. Our results also found generally better learning performance for non-reinforced test compared with reinforced training blocks, although Pavlovian bias was increased for those trials. Notably, this again emphasizes that Pavlovian bias does not by implication lead to poorer general learning performance. Our results indicate a similar shift in response strategy, as reported in Kurtenbach et al. (2022). While performance in nogo conditions was increased in test relative to training blocks, the opposite was true for GAL. Thus, performance differences between blocks and enhanced Pavlovian biases in test relative to training blocks may also be explained in terms of a reduced go bias, possibly due to a shift to a more cautious response style.

### Limitations and future directions

The present study has three main limitations. First, although the recruited participants were in a similar age range compared with previous studies (e.g. Cavanagh et al., 2013; Guitart-Masip et al., 2012; Peterburs et al., 2021), the results might not be representative. Crucially, Pavlovian bias has been shown to differently affect different age groups, with reduced Pavlovian bias reported for adolescents compared with children and young adults (Raab & Hartley, 2020), as well as a continuous decrease with age in adulthood (Chen et al., 2018). Thus, our study only captures a small section of participants, and it might be interesting to test in the future whether different age groups react differently to emotionality manipulations of the Pavlovian bias. Here, older adults might be of particular interest, given that ageing is associated with enhanced emotional processing as manifested in an attentional “positivity shift” (Carstensen & Mikels, 2005; Mather & Carstensen, 2003). Accordingly, one could speculate that using our task design in older individuals might yield a reduced or even absent Pavlovian bias.

Second, it has to be noted that in our task design, participants learned from facial cues but did not directly respond to the facial stimuli but to a neutral target stimulus (open white circle) after a short delay. Therefore, it might be possible that the observed effects underestimate the reported emotionality effects. Although Chiu et al. (2014) could show that cue valence can bias motor excitability and thus action tendencies even before action selection, future research should test if directly approaching versus avoiding facial cues has the potential to fully overcome the Pavlovian bias.

Third, our data provide evidence for reduced Pavlovian biases on instrumental learning, still whether this effect persists in a standard task remains unclear. Ereira et al. (2021) could show that a training-related reduction of Pavlovian biases even persisted in an independent task. Thus, it might be interesting to follow up whether the observed effects from the emotional variants can also be transferred to the standard version.

We believe that the present findings can have important implications for clinical practice. Pavlovian bias has been assumed to contribute to maladaptive behaviors including drug-seeking and addiction (Everitt et al., 2008; Flagel et al., 2010) as well as pathological gambling (Rutledge et al., 2015). Beyond that, altered Pavlovian bias has been linked to various neuropsychiatric conditions. For instance, the Pavlovian bias has been found to be increased in patients experiencing traumatic stress (Ousdal et al., 2018) and depression (Nord et al., 2018). For depression, an enhanced Pavlovian bias has been even considered predictive of recovery (Huys et al., 2016). Interestingly, reduced Pavlovian bias has been reported in schizophrenia, reflecting poorer learning performance in non-conflict conditions (Albrecht et al., 2016), similar to the result pattern in INC. Importantly, regulation of learning biases such as the Pavlovian bias may help overcome maladaptive behaviors. Our results show that the Pavlovian bias can be effectively reduced by increasing the cue-related arousal or cue salience through the use of social affective cues without the need of extensive training. Whether and how patients may benefit from better Pavlovian bias regulation should be addressed in future research.

## Conclusion

In conclusion, Pavlovian bias over instrumental behavior can be effectively reduced through learning from affective facial cues. This effect may be driven by increased cue arousal and/or salience. However, whether a reduced Pavlovian bias is adaptive and hence promotes goal-directed behavior, seems to be determined by cue valence, i.e., the congruency between the cue and action valence. This suggests that cue arousal/salience and cue valence may serve different aspects of learning. While cue arousal and salience may have the potential to improve Pavlovian regulation, cue valence seems to rather affect the adaptiveness related to goal-directed behavior. To our knowledge, our results constitute the first evidence of an easy-to-implement behavioral intervention resulting in reduced Pavlovian bias. However, the underlying mechanisms of this effect still remain unclear. Our findings might yield potential for clinical application in order to better understand how Pavlovian learning biases relate to psychiatric disorders.

## Electronic supplementary material

Below is the link to the electronic supplementary material.

Supplementary Material 1

## Data Availability

Data and code to reproduce all analyses in this manuscript can be found on the Open Science Framework (https://osf.io/ayp5x/).
