# Supplementary material for: Emotional cues reduce Pavlovian interference in feedback-based go and nogo learning
Source: Psychol Res. 2024 Mar 14;88(4):1212–30. doi: 10.1007/s00426-024-01946-9 (PMC11142951; doi:10.1007/s00426-024-01946-9)
Supplement: Supplementary file 1 — Supplementary Material 1 [file 426_2024_1946_MOESM1_ESM.docx]

**SUPPLEMENTARY MATERIAL**

**Emotional Cues Reduce Pavlovian Interference in Feedback-Based Go and Nogo Learning**

Julian Vahedi^1^, Annakarina Mundorf^2^, Christian Bellebaum^1^ & Jutta Peterburs^2^

^1^Faculty of Mathematics and Natural Sciences, Heinrich Heine University Düsseldorf, Düsseldorf, Germany

^2^Institute for Systems Medicine and Department of Human Medicine, MSH Medical School Hamburg, Germany

Address for correspondence:

Julian Vahedi, M.Sc. Psych.

Heinrich-Heine-University Düsseldorf

Institute for Experimental Psychology, Department of Biological Psychology

Faculty of Mathematics and Natural Sciences, Heinrich Heine University Düsseldorf,

Düsseldorf, Germany

Universitätsstr. 1, D-40225 Düsseldorf, Germany

E-Mail: [Julian.Vahedi@hhu.de](mailto:Julian.Vahedi@hhu.de)

**Table S1**

*Sample characteristics*

|  | CON (*n* = 43) | INC (*n* = 48) | NEU (*n* = 46) |
| --- | --- | --- | --- |
| Demographic characteristics |  |  |  |
| mean (*SD*) age in years | 22.12 (2.96) | 22.35 (2.96) | 22.54 (3.42) |
| sex (female/male), *n* | 29/14 | 33/15 | 33/13 |
| handedness (left/right/n.a.), *n* | 8/35/0 | 4/41/3 | 3/40/3 |
|  |  |  |  |
| Mean (*SD*) score |  |  |  |
| verbal IQ | 101.42 (11.32) | 102.38 (10.35) | 103.46 (10.79) |
| BIS | 20.53 (4.04) | 20.42 (4.21) | 20.46 (3.80) |
| BAS | 39.26 (5.74) | 41.42 (3.82) | 40.37 (4.25) |

Note: Demographic data and questionnaire scores are provided. Abbreviations are used as follows: CON = emotional-congruent group, INC = emotional-incongruent group, NEU = neutral group, SD = standard deviation, n.a. = not available, BIS = Behavioral Inhibition System, BAS = Behavioral Activation System.

# Supplementary Analyses

## Sensitivity Power Analysis

A sensitivity power analysis was conducted to identify the minimal effect size that can be detected with the current sample size for a desired statistical power of 80% at an α-level of .05. We opted for a simulation-based approach implemented in the *simr* package in R (Green & MacLeod, 2016). Specifically, the sensitivity power analysis was based on the three-way interaction effect between the factors learning condition, action, and valence of the fitted linear mixed-effects (LME) model for choice accuracy data. In the LME model, this three-way interaction is indicated by two separate contrasts, neutral (NEU) vs. congruent (CON) and NEU vs. incongruent (INC), for each of which an own (unstandardized) effect size estimate is provided in the model summary. Due to high computational cost in the simulation process, the sensitivity power analysis was solely based on the CON vs. NEU contrast of the three-way interaction. Therefore, we iteratively substituted the empirical β-coefficient with hypothetical unstandardized effect sizes ranging from 5 to 50 (step size = 5). Simulation-based sensitivity power analysis was then conducted using the powerSim() function for each of these hypothetical effect sizes in 250 simulations. Results revealed that for a desired statistical power of 80%, our study design with the current sample size is sensitive to detect an (unstandardized) effect size of 25.3 (see Figure S1). Observed, i.e., empirical (unstandardized) effect sizes after model fitting for both the NEU vs. CON (|β| = 28.67) and NEU vs. INC (|β| = 30.51) interaction contrast were larger than the minimal detectable effect size for a desired statistical power of 80% of 25.3.

**Figure S1**

*Sensitivity Power Analysis*


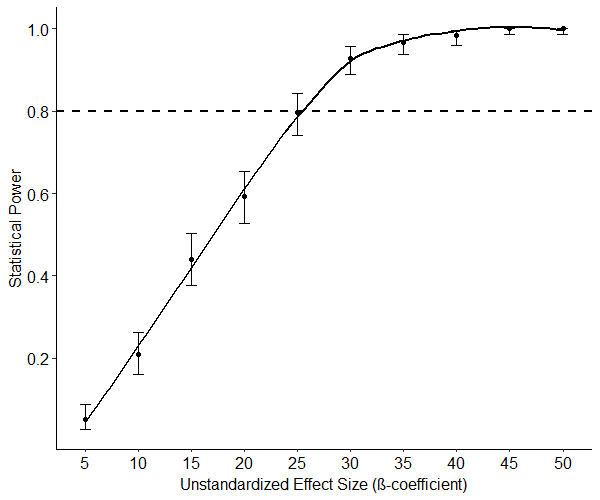


*Note*: Sensitivity power analysis was conducted for a range of hypothetical effect sizes for the three-way interaction effect between learning condition, action and valence. For a desired statistical power of 80%, results revealed a minimal detectable effect size of 25.3.

## Stimulus Rating

In order to assess whether the ratings on perceived facial expression, age, attractiveness, and distinctiveness differed between learning conditions, and to validate whether ratings obtained in the present sample were similar to ratings reported in Ebner et al. (2010) and Ebner et al. (2018), study participants were asked to rate the four facial stimuli that were presented during the experiment after task completion. Ratings were obtained on a scale ranging from 1 to 100, similar to Ebner et al. (2010) and Ebner et al. (2018). Participants rated one facial stimulus at a time for perceived facial expression (*How negative/positive is the facial expression of this person?*; 1 = very negative, 100 = very positive), age (*How old is this person*?; 1 = one year old, 100 = 100 years old), attractiveness (*How attractive is this person*?; 1 = not attractive at all, 100 = very attractive), and distinctiveness (*How distinctive is this person?*; 1 = not distinctive at all, 100 = very distinctive). Both facial cues and rating dimensions were presented in randomized order. Note that we report ratings only for participants after exclusions based on quality control criteria (*N* = 122).

Stimulus ratings were explored in four steps. First, we assessed whether ratings obtained for happy and angry facial stimuli differed between learning conditions: emotional-congruent (CON) and emotional-incongruent (INC). Therefore, we ran separate one-way analyses of variance (ANOVAs) for each rated emotional facial stimulus with the dependent variable set to perceived facial expression, age, and distinctiveness, respectively. Learning condition (CON, INC) was included as the between-subjects factor in each ANOVA model. There were no significant differences between learning conditions in any rating variable for any affective facial stimulus (all *p*s > .058).

Second, as a general manipulation check, we assessed whether valence ratings differed between happy, angry, and neutral facial stimuli by calculating a one-way ANOVA with the within-subjects factor affective valence (*positive, negative, neutral*) and the dependent variable perceived emotional expression. Not surprisingly, results revealed a significant main effect of affective valence, *F*(2, 481) = 650.3, *p* < .001, *η_p_^2^* = 0.73 (95%-CI 0.69-0.76). Post-hoc *t*-tests with false-discovery-rate (FDR) correction for multiple comparisons indicated that happy faces (*M* = 84.35, *SE* = 1.26) were rated as more positive than neutral (*M* = 45.79, *SE* = 1.24), *t*(481) = 21.82, *p* < .001, and angry faces (*M* = 20.71, *SE* = 1.26), *t*(481) = 35.79, *p* <.001, and that angry faces were rated as more negative than neutral faces, *t*(481) = 14.19, *p* < .001.

Third, we checked, whether facial stimuli were comparable regarding the perceived age, attractiveness, and distinctiveness, Here, we ran three separate one-way ANOVAs with the dependent variable set to perceived age, attractiveness, and distinctiveness, respectively. Again, affective valence (*positive*, *negative*, *neutral*) served as the within-subjects factor. There were significant differences indicated by a significant main effect of the factor affective valence for age, *F*(2, 481) = 19.51, *p* < .001, *η_p_^2^* = 0.08 (95%-CI 0.03-0.12), attractiveness, *F*(2, 481) = 8.00, *p* < .001, *η_p_^2^* = 0.03 (95%-CI 0.01-0.07), and distinctiveness, *F*(2, 481) = 8.50, *p* < .001, *η_p_^2^* = 0.03 (95%-CI 0.01-0.07). Post-hoc pairwise *t*-tests with FDR-correction for multiple comparisons indicated that age was generally rated as higher for angry (*M* = 29.48, *SE* = 0.39) relative to happy (*M* =26.05, *SE* = 0.39), *t*(481) = 6.23, *p* < .001, and neutral faces (*M* = 27.98, *SE* = 0.39), *t*(481) = 2.73, *p* < .001. In addition, age was rated as higher for neutral relative to happy faces, *t*(481) = 3.54, *p* = .001. Interestingly, happy faces (*M* = 46.65, *SE* = 1.77) were rated as less attractive than angry (*M* = 56.18, *SE* = 1.77), *t*(481) = 3.80, *p* < .001, and neutral faces (*M* = 54.13, *SE* = 1.75), *t*(481) = 3.00, *p* = .004. Distinctiveness was rated to be higher for angry (*M* = 61.47, *SE* = 1.75) compared to happy (*M* = 51.48, *SE* = 1.75), *t*(481) = 4.03, *p* < .001, and neutral faces (*M* = 54.66, *SE* = 1.73), *t*(481) = 2.77, *p* = .009.

To validate whether the obtained ratings were similar to ratings reported in Ebner et al. (2010) and Ebner et al. (2018), we calculated separate one-sample *t*-tests for the ratings of each facial stimulus against the corresponding mean rating obtained by Ebner et al. (2010) and Ebner et al. (2018) for age, attractiveness, and distinctiveness, respectively. Results are summarized in Table S2. Results indicated that ratings obtained from our sample deviated from those reported in Ebner et al. (2010) and Ebner et al. (2018). Most consistently, distinctiveness was rated significantly higher in our sample (all *p*s < .010). Age ratings also slightly but significantly differed between our ratings and those reported in Ebner et al. (2010) and Ebner et al. (2018) for all emotional facial stimuli and one neutral facial stimulus (*p*s < .001). Note however, that in the present study, ratings were obtained after completion of the experimental task, therefore individual learning success in the experimental conditions cued by the respective facial stimuli likely affected the ratings.

**Table S2**

*Stimulus Ratings*

| Filename | Valence | Mean (*SD*) rating value | Mean rating values obtained from Ebner et al. (2010) and Ebner et al. (2018) | *\|t\|* | *df* | *p*-value |
| --- | --- | --- | --- | --- | --- | --- |
| 182_y_f_h_b.jpg | positive | ***Age*** |  |  |  |  |
|  |  | **24.91 (4.84)** | **27.05** | **3.95** | **79** | **<.001***** |
|  |  | *Attractiveness* |  |  |  |  |
|  |  | 48.59 (20.37) | 51.20 | 1.15 | 79 | 0.255 |
|  |  | ***Distinctiveness*** |  |  |  |  |
|  |  | **48.56 (20.51)** | **37.29** | **4.92** | **79** | **<.001***** |
|  |  |  |  |  |  |  |
| 105_y_m_h_b.jpg | positive | ***Age*** |  |  |  |  |
|  |  | **27.19 (4.44)** | **29.48** | **4.62** | **79** | **<.001***** |
|  |  | *Attractiveness* |  |  |  |  |
|  |  | 44.71 (22.54) | 49.48 | 1.89 | 79 | .062 |
|  |  | ***Distinctiveness*** |  |  |  |  |
|  |  | **54.38 (23.25)** | **42.80** | **4.57** | **79** | **<.001***** |
|  |  |  |  |  |  |  |
| 098_y_f_a_b.jpg | negative | ***Age*** |  |  |  |  |
|  |  | **31.75 (4.61)** | **28.91** | **5.51** | **79** | **<.001***** |
|  |  | *Attractiveness* |  |  |  |  |
|  |  | 50.26 (19.85) | 50.25 | 0.11 | 79 | 0.991 |
|  |  | ***Distinctiveness*** |  |  |  |  |
|  |  | **59.86 (21.40)** | **38.10** | **9.10** | **79** | **<.001***** |
|  |  |  |  |  |  |  |
| 175_y_m_a_b.jpg | negative | ***Age*** |  |  |  |  |
|  |  | **27.2 (4.75)** | **29.19** | **3.75** | **79** | **<.001***** |
|  |  | ***Attractiveness*** |  |  |  |  |
|  |  | **62.08 (23.13)** | **41.54** | **7.94** | **79** | **<.001***** |
|  |  | ***Distinctiveness*** |  |  |  |  |
|  |  | **63.01 (20.28)** | **38.33** | **10.92** | **79** | **<.001***** |
|  |  |  |  |  |  |  |
| 182_y_f_n_b.jpg | neutral | *Age* |  |  |  |  |
|  |  | 25.22 (2.81) | 25.48 | 0.59 | 40 | .556 |
|  |  | *Attractiveness* |  |  |  |  |
|  |  | 53.46 (21.51) | 54.08 | 0.18 | 40 | .856 |
|  |  | ***Distinctiveness*** |  |  |  |  |
|  |  | **42.39 (20.39)** | **33.83** | **2.69** | **40** | **.010*** |
|  |  |  |  |  |  |  |
| 105_y_m_n_b.jpg | neutral | *Age* |  |  |  |  |
|  |  | 27.39 (4.04) | 26.92 | 0.74 | 40 | .461 |
|  |  | *Attractiveness* |  |  |  |  |
|  |  | 41.66 (20.88) | 39.13 | 0.78 | 40 | .442 |
|  |  | ***Distinctiveness*** |  |  |  |  |
|  |  | **45.83 (19.38)** | **29.19** | **5.50** | **40** | **<.001***** |
|  |  |  |  |  |  |  |
| 098_y_f_n_b.jpg | neutral | ***Age*** |  |  |  |  |
|  |  | **30.07 (4.73)** | **26.39** | **4.99** | **40** | **<.001***** |
|  |  | *Attractiveness* |  |  |  |  |
|  |  | 51.37 (21.44) | 55.01 | 1.09 | 40 | .283 |
|  |  | ***Distinctiveness*** |  |  |  |  |
|  |  | **61.41 (23.16)** | **40.05** | **5.91** | **40** | **<.001***** |
|  |  |  |  |  |  |  |
| 175_y_m_n_b.jpg | neutral | *Age* |  |  |  |  |
|  |  | 29.24 (5.70) | 29.73 | 0.55 | 40 | 0.588 |
|  |  | ***Attractiveness*** |  |  |  |  |
|  |  | **70.02 (21.47)** | **56.45** | **4.05** | **40** | **<.001***** |
|  |  | ***Distinctiveness*** |  |  |  |  |
|  |  | **69.00 (20.62)** | **42.70** | **8.17** | **40** | **<.001***** |
|  |  |  |  |  |  |  |

*Note*: SD = standard deviation, df = degrees of freedom

*p < .05, **p < .01, ***p < .001

## Linear Mixed-Effects Model Analysis of Response Time Data

Analysis of response times (RTs) was only performed for correct go trials. We fitted a LME model similar to the choice accuracy model but excluding the factor action. Visual inspection of the RT distribution indicated non-normality, so RTs were first log-transformed before entered as the dependent variable into the LME model. The full model with a maximal random-effects structure resulted in a singular fit, therefore we simplified the model manually via stepwise elimination of random effects until convergence was reached. The simplified LME model for RT data was specified as follows:

$$log(RT) \sim learning condition*valence*block type*block+\left( 1+valence+block type+block \right| participant)$$

No participants exceeded the Cook’s distance criterion, thus data from 122 participants were used for the statistical analysis of RT data.

Most importantly, the LME model analysis revealed a significant main effect of valence, *F*(1,125.04) = 80.57, *p* < .001, *η_p_^2^* = 0.39 (95%-CI 0.26-0.50), indicating increased RTs for avoid-losing relative to win, β = 0.08 (*SE* = 0.01). In addition, there was a main effect of block type, *F*(1,118.17) = 26.51, *p* < .001, *η_p_^2^* = 0.18 (95%-CI 0.07-0.31), with generally slower RTs for training relative to test blocks, β = -0.04 (*SE* = 0.01). Additionally, we found a significant main effect of block, *F*(1,119.01) = 9.98,  *p* = .002, *η_p_^2^* = 0.08 (95%-CI 0.01-0.18), indicating that overall RTs decreased over experimental blocks, β = - 0.1 (*SE* < 0.01). Last, results revealed a significant learning condition × block type × block interaction effect, *F*(2,1438.74) = 3.97, *p* = .020, *η_p_^2^* = 0.01 (95%-CI 0-0.01). Resolving this interaction effect separately for learning conditions indicated that RTs decreased across both training, β = -0.03 (*SE* = 0.01), *p* = .015, FDR-corrected, and test blocks, β = -0.02 (*SE* = 0.01), *p* = .004, FDR-corrected, in NEU. In contrast, RTs decreased only across test, β = -0.02 (*SE* = 0.01), *p* = .041, FDR-corrected, but not training block, *p* = .363, FDR-corrected, in CON. No such response time speeding neither across training, *p* = .399, FDR-corrected, nor test blocks, *p* = .399, FDR-corrected, was observed in INC. The inferential statistics for all fixed effects of the LME model can be found in Table S3.

**Table S3**

*Regression-table for the fixed effects of the linear-mixed effects model for response time data*

| Fixed Effect | β | SE | df | *F*/*t* | *p* |
| --- | --- | --- | --- | --- | --- |
| ***(intercept)*** | ***6.17*** | ***0.01*** | ***119.60*** | ***520.45*** | ***<.001****** |
| learning condition |  |  | 2, 119.61 | 0.29 | .745 |
| *NEU vs. CON* | *0.02* | *0.03* | *119.50* | *0.67* | *.505* |
| *NEU vs. INC* | *<.01* | *0.03* | *119.70* | *0.01* | *.996* |
| **valence** | **0.08** | **0.01** | **1, 125.04** | **80.57** | **<.001***** |
| **block type** | **-0.04** | **0.01** | **1, 118.17** | **26.51** | **<.001***** |
| **block** | **-0.01** | **<.01** | **1, 119.01** | **9.68** | **.002**** |
| learning condition × valence |  |  | 2, 125.04 | 0.48 | .622 |
| *NEU vs. CON* | *-0.01* | *0.02* | *124.40* | *-0.28* | *.782* |
| *NEU vs. INC* | *-0.02* | *0.02* | *125.80* | *-0.95* | *.344* |
| learning condition × block type |  |  | 2, 118.17 | 0.83 | .441 |
| *NEU vs. CON* | *<.01* | *0.02* | *117.40* | *-0.25* | *.803* |
| *NEU vs. INC* | *-0.02* | *0.02* | *119.20* | *-1.22* | *.226* |
| valence × block type | 0.02 | 0.01 | 1, 1438.81 | 3.19 | .074 |
| learning condition × block |  |  | 2, 119.02 | 1.80 | .169 |
| *NEU vs. CON* | *0.02* | *0.01* | *118.40* | *1.75* | *.083* |
| *NEU vs. INC* | *0.02* | *0.01* | *119.70* | *1.51* | *.134* |
| valence × block | <.01 | 0.01 | 1, 1434.37 | 0.25 | .616 |
| block type × block | -0.01 | 0.01 | 1, 1438.76 | 1.45 | .228 |
| learning condition × valence × blockType |  |  | 2, 1438.79 | 0.17 | .840 |
| *NEU vs. CON* | *-0.02* | *0.03* | *1433.00* | *-0.53* | *.597* |
| *NEU vs. INC* | *-0.01* | *0.03* | *1443.00* | *-0.49* | *.624* |
| learning condition × valence × block |  |  | 2, 1434.37 | 2.29 | .102 |
| *NEU vs. CON* | *<.01* | *0.02* | *1433.00* | *0.27* | *.784* |
| ***NEU vs. INC*** | ***0.03*** | ***0.02*** | ***1437.00*** | ***1.98*** | ***.048**** |
| **learning condition × block type × block** |  |  | **2, 1438.74** | **3.97** | **.019*** |
| ***NEU vs. CON*** | ***-0.04*** | ***0.02*** | ***1433.00*** | ***-2.56*** | ***.011**** |
| *NEU vs. INC* | *<.01* | *0.02* | *1443.00* | *-0.24* | *.811* |
| valence × block type × block | 0.01 | 0.01 | 1, 1434.5 | 1.13 | .287 |
| learning condition × valence × block type × block |  |  | 2, 1434.5 | 0.23 | .797 |
| *NEU vs. CON* | *<.01* | *0.03* | *1433.00* | *-0.09* | *.929* |
| *NEU vs. INC* | *-0.02* | *0.03* | *1437.00* | *-0.62* | *.533* |

*Note:* *SE*=standard error, df = degrees of freedom; *t*-statistic rather than *F*-statistic is provided for contrasts related to the three-level predictor learning condition (corresponding rows are italicized).

*p < .05, **p < .01, ***p < .001

## References

Ebner, N. C., Luedicke, J., Voelkle, M. C., Riediger, M., Lin, T., & Lindenberger, U. (2018). An adult developmental approach to perceived facial attractiveness and distinctiveness. *Frontiers in psychology, 9*, 561.

Ebner, N. C., Riediger, M., & Lindenberger, U. (2010). FACES—A database of facial expressions in young, middle-aged, and older women and men: Development and validation. *Behavior research methods, 42*(1), 351-362.

Green, P., & MacLeod, C. J. (2016). SIMR: An R package for power analysis of generalized linear mixed models by simulation. *Methods in Ecology and Evolution, 7*(4), 493-498.
